# Efficacy of Climate Forcings in PDRMIP Models

**DOI:** 10.1029/2019JD030581

**Published:** 2019-12-11

**Authors:** T. B. Richardson, P. M. Forster, C. J. Smith, A. C. Maycock, T. Wood, T. Andrews, O. Boucher, G. Faluvegi, D. Fläschner, Ø. Hodnebrog, M. Kasoar, A. Kirkevåg, J.‐F. Lamarque, J. Mülmenstädt, G. Myhre, D. Olivié, R. W. Portmann, B. H. Samset, D. Shawki, D. Shindell, P. Stier, T. Takemura, A. Voulgarakis, D. Watson‐Parris

**Affiliations:** ^1^ Priestley International Centre for Climate University of Leeds Leeds UK; ^2^ Met Office Hadley Centre Exeter UK; ^3^ Institut Pierre‐Simon Laplace CNRS/Sorbonne Université Paris France; ^4^ NASA Goddard Institute for Space Studies and Center for Climate Systems Research Columbia University New York NY USA; ^5^ Atmosphere in the Earth System Max‐Planck‐Institut für Meteorologie Hamburg Germany; ^6^ CICERO Center for International Climate and Environmental Research Oslo Norway; ^7^ Department of Physics Imperial College London London UK; ^8^ Research and Development Department Norwegian Meteorological Institute Oslo Norway; ^9^ NCAR/UCAR Boulder CO USA; ^10^ Clouds and Global Climate Universität Leipzig Leipzig Germany; ^11^ Earth System Research Laboratory National Oceanic and Atmospheric Administration Boulder CO USA; ^12^ Earth & Ocean Sciences Duke University Durham NC USA; ^13^ Atmospheric, Oceanic and Planetary Physics, Department of Physics University of Oxford Oxford UK; ^14^ Center for Oceanic and Atmospheric Research Kyushu University Fukuoka Japan

**Keywords:** Efficacy, Climate Sensitivity, Radiative Forcing, Surface temperature, PDRMIP

## Abstract

Quantifying the efficacy of different climate forcings is important for understanding the real‐world climate sensitivity. This study presents a systematic multimodel analysis of different climate driver efficacies using simulations from the Precipitation Driver and Response Model Intercomparison Project (PDRMIP). Efficacies calculated from instantaneous radiative forcing deviate considerably from unity across forcing agents and models. Effective radiative forcing (ERF) is a better predictor of global mean near‐surface air temperature (GSAT) change. Efficacies are closest to one when ERF is computed using fixed sea surface temperature experiments and adjusted for land surface temperature changes using radiative kernels. Multimodel mean efficacies based on ERF are close to one for global perturbations of methane, sulfate, black carbon, and insolation, but there is notable intermodel spread. We do not find robust evidence that the geographic location of sulfate aerosol affects its efficacy. GSAT is found to respond more slowly to aerosol forcing than CO_2_ in the early stages of simulations. Despite these differences, we find that there is no evidence for an efficacy effect on historical GSAT trend estimates based on simulations with an impulse response model, nor on the resulting estimates of climate sensitivity derived from the historical period. However, the considerable intermodel spread in the computed efficacies means that we cannot rule out an efficacy‐induced bias of ±0.4 K in equilibrium climate sensitivity to CO_2_ doubling when estimated using the historical GSAT trend.

## Key Points

- Multimodel mean efficacies computed using effective radiative forcing are close to one for major anthropogenic drivers, but there is notable intermodel spread
- Surface temperature‐driven radiative feedbacks are generally not constant through time across forcing experiments
- PDRMIP results suggest that the efficacy impact on equilibrium climate sensitivity derived from the historical period is limited to ±0.4 °C or better

## 1 Introduction

Quantifying the effectiveness of different physical drivers in altering surface temperature is important for understanding historical and future temperature trends (Kummer & Dessler, 2014; Marvel et al., 2015). Global mean near‐surface air temperature (GSAT) change is typically understood in terms of the well‐established radiative forcing and response framework (equation (1); Hansen et al., 1997; Boer & Yu, 2003; Gregory, 2004; Myhre et al., 2013). Radiative forcing represents the change in the Earth's energy balance due to an imposed perturbation, such as increasing greenhouse gases. In response to an imposed forcing which is held constant, GSAT will increase or decrease until the climate system reaches a new equilibrium. The temperature response depends on both the magnitude of the initial forcing and the temperature‐driven radiative feedbacks, which are represented by the climate feedback parameter (*α*) in equation (1).

The usefulness of radiative forcing as a predictor of GSAT change is dependent on the climate feedback parameter being the same across different forcing agents or equivalently that the “efficacy” of a forcing agent is close to unity. We define efficacy as the relative change in GSAT induced by a forcing agent compared to carbon dioxide, for an equivalent perturbation in global mean energy balance at the top of the atmosphere (TOA) or tropopause. Previous studies have indicated that the efficacies of different forcing agents may vary significantly from unity (Hansen et al., 2005; Manabe & Stouffer, 1980; Marvel et al., 2015; Modak et al., 2016; Modak et al., 2018; Modak & Bala, 2019; Shindell, 2014; Shindell et al., 2015; Stuber et al., 2005). Hansen et al. (2005) showed that in a single model, efficacies varied widely across many forcing agents, identifying an efficacy of ~1.5 for methane and ~0.6 for black carbon (BC). Several studies have also found that solar forcing has an efficacy of less than one (Forster et al., 2000; Hansen et al., 2005; Modak et al., 2016; Schmidt et al., 2012).

An important factor to consider is that efficacies are dependent on the method by which radiative forcing is defined (Hansen et al., 2005). Traditionally, radiative forcing was calculated at the tropopause as an instantaneous radiative forcing (IRF) or by accounting for stratospheric temperature adjustment (RF; Forster et al., 2007; Ramaswamy et al., 2001). However, these methods do not account for rapid adjustments which occur in the troposphere, such as changes to temperature, water vapor, and clouds (Andrews & Forster, 2008; Colman & McAvaney, 2011; Gregory & Webb, 2008; Kamae & Watanabe, 2013). The Intergovernmental Panel on Climate Change Fifth Assessment Report were able to estimate effective radiative forcing (ERF) for a few forcing agents, where ERF accounts for rapid adjustments of both stratospheric and tropospheric parameters (Boucher et al., 2013; Myhre et al., 2013). The ERF framework provides a more complete understanding of the energy budget response to different drivers than more traditional radiative forcing definitions (Hansen et al., 2005; Sherwood et al., 2015) and accounts for some of the differences between GSAT responses to different drivers that were previously accounted for by employing nonunity efficacies. However, some studies using ERF still find efficacies deviating from unity (Marvel et al., 2015; Modak et al., 2018, 2016; Modak & Bala, 2019; Shindell et al., 2015). This is further complicated by the fact that there are various methods for calculating ERF in models, which could affect efficacies (Forster et al., 2016; Sherwood et al., 2015).

Recent studies using the ERF framework to analyze transient historical simulations suggest that tropospheric aerosols drive a stronger transient climate response than carbon dioxide by around 50% (Shindell, 2014; Shindell et al., 2015). This has been mainly attributed to the inhomogeneous nature of aerosol forcing, which over the historical period has been more concentrated in the Northern Hemisphere extratropics, where there is evidence from models for a stronger sensitivity of temperature to localized forcing (Shindell, 2014; Shindell et al., 2015). As historical aerosol forcing is negative, this suggests that the higher efficacy could in part account for a lower than expected observed GSAT change and climate sensitivity derived from the Earth's energy budget (Forster, 2016; Knutti et al., 2017; Shindell et al., 2012). Accounting for different forcing efficacies could significantly increase climate sensitivity estimates from the historical period (Kummer & Dessler, 2014; Marvel et al., 2015). Therefore, quantifying forcing efficacies and understanding how robust they are across different models are of significant importance.

Only the Goddard Institute for Space Studies (GISS) modeling center has systematically diagnosed forcings and efficacies from their climate model (Hansen et al., 2005; Marvel et al., 2015). The lack of results from other models makes it difficult to test the robustness of the earlier findings and determine the implications for the real world. In this study we make use of the Precipitation Driver and Response Model Intercomparison Project (PDRMIP) data set (Myhre et al., 2017) to perform a systematic multimodel analysis of forcing efficacies. We compute efficacies from idealized single forcing simulations for a range of drivers across 11 PDRMIP models. In addition, we examine how the location of aerosol forcing affects efficacies. We compute radiative forcing using multiple methods to better understand the robustness of earlier findings and the dependence of efficacy calculations on radiative forcing definition as the method of radiative forcing calculation with the least efficacy variation could become the determining factor in defining forcing (Hansen et al., 2005).

## 2 Methods

### 2.1 Models and Simulations

We analyze output from 11 global climate models (see supporting information Table S1 for model details) participating in PDRMIP (Myhre et al., 2017). PDRMIP simulations are designed as idealized single forcing experiments to probe the multimodel robustness of responses to individual forcings and understand the role of rapid adjustments. Simulations were performed for five core global single forcing perturbations: doubling CO_2_ concentration (2×CO2), tripling methane concentration (3×CH4), five times sulfate aerosol concentrations or emissions (5×SO4), ten times BC aerosol concentrations or emissions (10×BC), and a two‐percent increase in insolation (2%SOL). An additional nine simulations were performed by a subset of models, to investigate the effect of regional forcings and further forcing agents, as detailed in Table S2. The aerosol and ozone forcing experiments have spatially varying concentrations. The perturbations are made relative to either present‐day or pre‐industrial baseline conditions (see Table S1). Simulations were performed with a coupled ocean for 100 years. Two models (ECHAM‐HAM and CESM‐CAM4) used a slab ocean, and these results are excluded from multimodel mean calculations of response as they do not allow for ocean circulation changes. An additional set of 15 years of simulations were performed with sea surface temperatures and sea‐ice fixed (fSST) at the present‐day or pre‐industrial model climatology in atmospheric and land surface versions of the coupled and slab models to estimate forcings. All responses are calculated by subtracting the control run from the corresponding perturbed run.

Note that the experiments were performed by models that both prescribe concentrations of greenhouse gases and aerosols and models that prescribe greenhouse gases but use emissions derived aerosol concentrations (Table S1). In model configurations that perturbed emissions, the atmospheric distribution of aerosols is determined by the models own aerosol and cloud schemes, which introduces an additional element of uncertainty that is not present in the experiments in which atmospheric concentrations are prescribed. The models also adopt different treatments of aerosol microphysics and/or stratospheric water vapor oxidation (Table S1). None of the models considered include full interactive chemistry. One model (MPI‐ESM) did not include aerosols and hence did not perform the aerosol perturbation experiments. CESM‐CAM4 and GISS excluded aerosol microphysics changes to clouds (the Twomey effect). Overall, the differing model setups mean that aerosol concentrations, and hence forcings, varied across the suite of models and this should be kept in mind when evaluating the spread of model responses.

### 2.2 Forcing and Response

The energy balance of the climate system can be described using a simple linear equation relating radiative forcing and response (e.g., Boer & Yu, 2003; Gregory, 2004; Hansen et al., 1981):

(1) N=F−α∆T,

where the net radiative imbalance at the TOA (*N*) due to a driver is equal to the radiative forcing (*F*) minus the radiative response (*αΔT*) which scales with GSAT change (*ΔT*) and “*α*” denotes the climate feedback parameter in W m^−2^/K.

### 2.3 Forcing Definitions

There are various commonly used radiative forcing definitions which depend on the method used for separating forcing and response. In this study we compute six different radiative forcing estimates, as detailed below, which each account for different degrees of atmospheric and land surface adjustment.

#### 2.3.1 Instantaneous Radiative Forcing (*IRF*_*toa*_ and *IRF*_*trop*_)

IRF at the TOA (*IRF*_*toa*_) is calculated for the five core PDRMIP experiments. IRF is also calculated at the tropopause (*IRF*_*trop*_) for 2×CO2 and 3×CH4. *IRF*_*toa*_ is calculated using the difference of the perturbed and base runs using a “double‐call” to the radiation code for 5×SO4 (available in three models) and 10×BC (six models). In each case an additional call to the radiation code is performed with the aerosol species of interest removed, and the *IRF*_*toa*_ is taken as the difference of the full‐aerosol and no‐aerosol calls in the perturbed run minus the difference of the full‐aerosol and no‐aerosol calls in the control run. For 2×CO2 and 3×CH4, *IRF*_*toa*_ and *IRF*_*trop*_ are estimated by substituting each model's control climatology into the SOCRATES radiation code (Edwards & Slingo, 1996) and taking the difference of radiative fluxes at the TOA or tropopause between the perturbed and control greenhouse gas concentrations in each model. For 2%SOL, *IRF*_*toa*_ is calculated as 2% × *SC* × (1 − *A*) /4, where *SC* is each model's solar constant and *A* is the control run planetary albedo.

#### 2.3.2 Stratospheric Temperature Adjusted Radiative Forcing (*RF*_*strat*_)

The stratospheric temperature adjusted radiative forcing is calculated by subtracting all rapid adjustments from *ERF*_*sst*_ (described below) except for stratospheric temperature change. The individual rapid adjustments are computed using the radiative kernel method outlined by Smith, Kramer, et al. (2018) and account for adjustments in tropospheric temperature, surface temperature, water vapor, surface albedo, and clouds.

#### 2.3.3 Fixed SST Effective Radiative Forcing (*ERF*_*sst*_)

We compute *ERF*_*sst*_ as the change in TOA radiative flux between the perturbed and control fSST simulations (Hansen et al., 2005). In these simulations SST‐driven radiative feedbacks are inhibited. *ERF*_*sst*_ therefore includes rapid adjustments of the troposphere, stratosphere, and land surface.

#### 2.3.4 Adjusted Effective Radiative Forcing (*ERF*_*ssta*_)

*ERF*_*ssta*_ is an “adjusted” version of *ERF*_*sst*_ which accounts for the land surface temperature changes that occur in the fSST simulations. The effect of land surface temperature change on the TOA energy balance is computed using radiative kernels and subtracted from *ERF*_*sst*_. An alternative method to account for the land surface temperature change is to adjust *ERF*_*sst*_ based on the climate feedback parameter computed from the coupled runs (Sherwood et al., 2015). This method assumes that the climate feedback parameter is the same when only land temperatures are increasing. Here both methods produce similar results, so only the kernel results are shown for brevity.

#### 2.3.5 Regression Effective Radiative Forcing (*ERF*_*reg*_)

Following equation (1), *ERF*_*reg*_ is calculated using linear regression of global mean TOA flux (*N*) change against GSAT change (*ΔT*) relative to the baseline simulation for the first 20 years of the coupled simulations. *ERF*_*reg*_ is given by the intercept of the regression line where *ΔT* = 0 (Gregory, 2004).

### 2.4 Efficacy Computation

#### 2.4.1 Efficacy Based on Forcing

We compute forcing efficacy by dividing the temperature response in the coupled PDRMIP simulations by the radiative forcing, as defined above, and normalizing with respect to the CO_2_ response:

(2) $$E_{f}=\frac{∆T/F}{{∆T}_{2\times \mathrm{CO}2}/F_{2\times \mathrm{CO}2}},$$

where *E*_*f*_ is the efficacy of a given forcing agent, *ΔT* is the GSAT response to the global mean forcing (*F*), and *ΔT*_2×CO2_ is the GSAT response to CO_2_ global mean forcing (*F*_2×CO2_). The GSAT responses are taken as the mean difference between the perturbed and baseline coupled PDRMIP simulations for years 81–100 of simulations. We calculate efficacies using the six different forcing definitions described above, denoting the efficacies *E*_*irf_toa*_, *E*_*irf_trop*_, *E*_*rf_strat*_, *E*_*erf_sst*_, *E*_*erf_ssta*_, and *E*_*erf_reg*_ (corresponding to the forcing definitions *IRF*_*toa*_, *IRF*_*trop*_, *RF*_*strat*_, *ERF*_*sst*_, *ERF*_*ssta*_, and *ERF*_*reg*_, respectively).

This definition of efficacy shows the effectiveness of climate drivers at changing GSAT on a centennial timescale similar to Hansen et al. (2005). We adopt this approach as the derived efficacies are more suited to analysis of long‐term historical temperature change, which is the focus of this work. However, it differs from the methods used in some studies where efficacies are computed from transient historical simulations (Marvel et al., 2015; Shindell, 2014). We also tested a calculation of efficacies more akin to equilibrium conditions by estimating the long‐term equilibrium temperature response from the flux imbalance toward the end of the model integration. Such efficacies differed by less than 0.05 compared to those presented here (or less than 0.1 for 10×BC integrations), with no clear systematic difference.

For each model, 5–95% confidence intervals are calculated based on the control run interannual variability. Confidence intervals (CIs) for ERF and *ΔT* are computed using equation (3):

(3) $$\mathrm{CI}=\frac{s}{\sqrt{n}}\times t_{n-1}\times \sqrt{2},$$

where *s* is the standard deviation of the annual means in the control run for the given variable, *n* is the number of years, *t*_*n* − 1_ is the corresponding *t* value from the *t* table with *n* − 1 degrees of freedom. The ERF and *ΔT* confidence intervals are then propagated through equation (2).

#### 2.4.2 Efficacy Based on Feedback Parameter

In addition to the calculation of efficacies based on GSAT and radiative forcing, we also compute forcing efficacies using the climate feedback parameter. The climate feedback parameter is calculated using linear regression of global mean TOA radiative flux change against GSAT change in the coupled PDRMIP simulations. A higher feedback parameter is associated with a smaller climate sensitivity, as less GSAT change is required to restore the climate system to equilibrium. Forcing efficacies based on the climate feedback parameter can then be computed using equation (4):

(4) $$E_{\alpha }=\frac{\alpha_{\mathrm{CO}2}}{\alpha_{f}},$$

where *E*_*α*_ is the forcing efficacy, *α*_CO2_ is the climate feedback parameter in response to CO_2_, and *α*_*f*_ is the climate feedback parameter in response to a given forcing. This efficacy definition implicitly assumes that the climate feedback parameter is constant through time.

### 2.5 Simple Model

We construct a simple impulse response model to estimate historical GSAT changes based on TOA forcing and PDRMIP GSAT response curves for the different drivers. The GSAT responses across the duration of the PDRMIP integrations were normalized by *ERF*_*sst*_ for each model and the multimodel mean computed. Following Larson and Portmann (2016), the normalized GSAT responses (*R*) are well fit using a two‐term exponential decay function of the form:

(5) $$R\left(t\right)=A\left(1-e^{-t/\tau_{1}}\right)+B\left(1-e^{-t/\tau_{2}}\right),$$

where *A* and *B* are amplitudes, *t* is time, and *τ*_1_ and *τ*_2_ are time constants. The GSAT change over the historical period due to a given forcing agent can then be calculated using

(6) $$∆T_{i}=\sum_{j=0}^{i}R_{i-j}\left(F_{j}-F_{j-1}\right),$$

where *∆T*_*i*_ is the change in GSAT in year *i*, *F*_*j*_ and *F*_*j* − 1_ are the global mean TOA forcing in year *j* and *j* − 1, and *R*_*i* − *j*_ is the discretized response curve where *t = i* − *j*. The total historical GSAT change time series is then computed using equation (6) for each historical forcing and summing the results.

Equation (6) treats each annual forcing change as initiating a separate GSAT impulse response going forward in time and then sums the responses together with those from past years to give a GSAT change through time associated with a given forcing agent. Distinct GSAT response curves are used for historical forcing from CO_2_, CH_4_, insolation, and aerosols (attributed 5×SO4 response curve). Since PDRMIP did not perform a volcanic perturbation experiment, the effect of volcanic aerosol is attributed to the 2%SOL response curve. All remaining historical forcings are attributed the 2×CO2 response curve. The historical forcing time series is an updated version of data from Myhre et al. (2013), see Dessler and Forster (2018) for details (http://climexp.knmi.nl/selectindex.cgi#ERF). The inclusion of volcanic episodic forcing within an impulse response framework is known to be imperfect (Gregory et al. 2016). Volcanoes are included here only to more or less represent the historic temperature trend in the impulse response model; their efficacy impacts are not examined in this work. Nevertheless, our multiforcing impulse response approach represents a considerable advance over previous work that assumed that all radiative forcings generally have the same global temperature response as CO_2_ (Good et al., 2013; Smith, Forster, et al., 2018).

## 3 Results and Discussion

### 3.1 Radiative Forcing of Core PDRMIP Experiments

**Figure 1:**
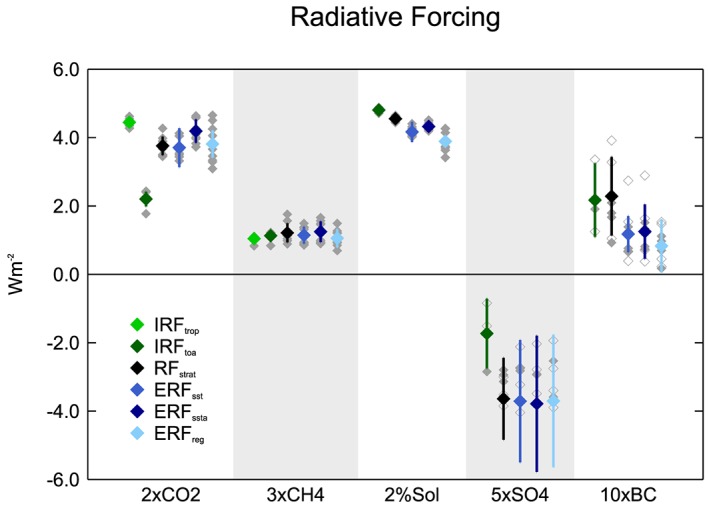
*IRF*_*trop*_, *IRF*_*toa*_, *RF*_*strat*_, *ERF*_*reg*_, *ERF*_*sst*_, and *ERF*_*ssta*_ for the five core PDRMIP forcings. Colored diamonds denote the multimodel mean, and error bars show the intermodel standard deviation. Individual model results are represented by gray diamonds. Hollow gray diamonds denote models which perturbed emissions rather than concentrations in the aerosol experiments. One model (HadGEM3) is off the bottom of the scale for 5×SO4.

We first compare the different radiative forcing definitions across the five core PDRMIP experiments (Figure 1 and Table 1). For the multimodel mean, the three ERF definitions are broadly consistent in magnitude. *ERF*_*ssta*_ is systematically slightly greater in magnitude than *ERF*_*sst*_ and *ERF*_*reg*_. Adjusting *ERF*_*sst*_ to account for the radiative feedbacks driven by land surface temperature change does not bring the results into closer agreement with *ERF*_*reg*_, as noted in previous studies (Hansen et al., 2005; Sherwood et al., 2015).

**Table 1:** PDRMIP Multimodel Mean Radiative Forcing (See section 2.3 for Definitions), GSAT Response, Climate Feedback Parameter Calculated Using First 20 years (*α*_20), Final 80 years (*α*_80), and Full 100 years (*α*) of Simulations, and Efficacies (See section 2.4 for Definitions) for the Five Core Experiments

	2×CO2	3×CH4	2%SOL	5×SO4	10×BC
*IRF*_*trop*_ (W m^−2^)	4.45 ± 0.11	1.04 ± 0.09	—	—	—
*IRF*_*toa*_ (W m^−2^)	2.20 ± 0.22	1.13 ± 0.13	4.81 ± 0.05	−1.73 ± 1.02	2.17 ± 1.08
*RF*_*strat*_ (W m^−2^)	3.76 ± 0.27	1.21 ± 0.28	4.55 ± 0.06	−3.64 ± 1.20	2.29 ± 1.15
*ERF*_*sst*_ (W m^−2^)	3.71 ± 0.30	1.15 ± 0.25	4.17 ± 0.13	−3.71 ± 1.94	1.18 ± 0.75
*ERF*_*ssta*_ (W m^−2^)	4.19 ± 0.35	1.25 ± 0.31	4.32 ± 0.12	−3.78 ± 1.99	1.25 ± 0.80
*ERF*_*reg*_ (W m^−2^)	3.81 ± 0.57	1.06 ± 0.25	3.89 ± 0.29	−3.70 ± 1.79	0.83 ± 0.53
Δ*T* (K)	2.44 ± 0.75	0.67 ± 0.33	2.46 ± 0.97	−2.45 ± 1.85	0.74 ± 0.54
*α* (W m^−2^/K)	−1.19 ± 0.50	−1.06 ± 0.53	−1.24 ± 0.51	−1.11 ± 0.34	−0.89 ± 0.46
*α*_20 (W m^−2^/K)	−1.27 ± 0.52	−1.28 ± 0.57	−1.36 ± 0.51	−1.51 ± 0.85	−0.97 ± 0.77
*α*_80 (W m^−2^/K)	−1.03 ± 0.57	−0.93 ± 0.73	−1.04 ± 0.51	−0.80 ± 0.36	−0.78 ± 0.60
Δ*T*/*IRF*_*trop*_ (K/W m^−2^)	0.55 ± 0.19	0.61 ± 0.33	—	—	—
Δ*T*/*IRF*_*toa*_ (K/W m^−2^)	1.14 ± 0.44	0.57 ± 0.35	0.52 ± 0.21	1.25 ± 0.81	0.25 ± 0.21
Δ*T*/*RF*_*strat*_ (K/W m^−2^)	0.66 ± 0.24	0.57 ± 0.29	0.54 ± 0.21	0.66 ± 0.25	0.40 ± 0.25
Δ*T*/*ERF*_*sst*_ (K/W m^−2^)	0.67 ± 0.22	0.58 ± 0.23	0.59 ± 0.23	0.62 ± 0.19	0.63 ± 0.37
Δ*T*/*ERF*_*ssta*_ (K/W m^−2^)	0.59 ± 0.19	0.54 ± 0.22	0.57 ± 0.22	0.61 ± 0.18	0.60 ± 0.34
Δ*T*/*ERF*_*reg*_ (K/W m^−2^)	0.66 ± 0.25	0.66 ± 0.32	0.63 ± 0.25	0.63 ± 0.22	1.22 ± 1.45
*E*_*irf_trop*_		1.09 ± 0.35	0.89 ± 0.09	2.97 ± 1.86	0.54 ± 0.29
*E*_*irf_toa*_	—	0.48 ± 0.17	0.44 ± 0.04	1.50 ± 0.99	0.27 ± 0.13
*E*_*rf_strat*_	—	0.84 ± 0.21	0.81 ± 0.06	0.99 ± 0.09	0.55 ± 0.16
*E*_*erf_sst*_	—	0.87 ± 0.15	0.87 ± 0.07	0.94 ± 0.16	0.87 ± 0.31
*E*_*erf_ssta*_	—	0.91 ± 0.18	0.95 ± 0.07	1.04 ± 0.16	0.93 ± 0.32
*E*_*erf_reg*_	—	0.97 ± 0.23	0.96 ± 0.09	0.95 ± 0.25	1.48 ± 1.09
*E*_*α*_	—	1.22 ± 0.47	0.97 ± 0.12	1.01 ± 0.29	1.36 ± 0.26

*Note*. Uncertainty bounds are the standard deviation of the intermodel spread.

*IRF*_*toa*_ is considerably smaller in magnitude than ERF for 2×CO2 and 5×SO4 and greater in magnitude for 2%SOL and 10×BC. These differences are a result of rapid adjustments of the stratosphere and troposphere (detailed in Smith, Kramer, et al., 2018), which are accounted for in ERF. For 2×CO2, stratospheric temperature adjustment is the main factor driving the larger ERF, and therefore, the stratospheric temperature adjusted *RF*_*strat*_ is in close agreement with ERF for 2×CO2. Tropospheric adjustments have a greater effect on ERF for 2%SOL and 10×BC. Large adjustments in tropospheric temperature and clouds result in a much smaller 10×BC ERF than *RF*_*strat*_ (Smith, Kramer, et al., 2018; Stjern et al., 2017).

The intermodel spread in radiative forcing is largest for the aerosol perturbations, which is partly due to the mix of concentration‐ and emission‐based perturbations, with emissions‐based scaling factors giving a range of concentrations, depending on the respective aerosol lifetimes in the models. HadGEM3 is a significant outlier for 5×SO4 with ERF ranging from −7.8 to −8.5 W m^−2^ depending on the calculation method. The 2%SOL and 3×CH4 experiments exhibit the smallest intermodel spread in radiative forcing. It should be noted that 3×CH4 and 10×BC produce smaller radiative forcing values than the other experiments, which increases the uncertainty of the calculated efficacies.

**Figure 2:**
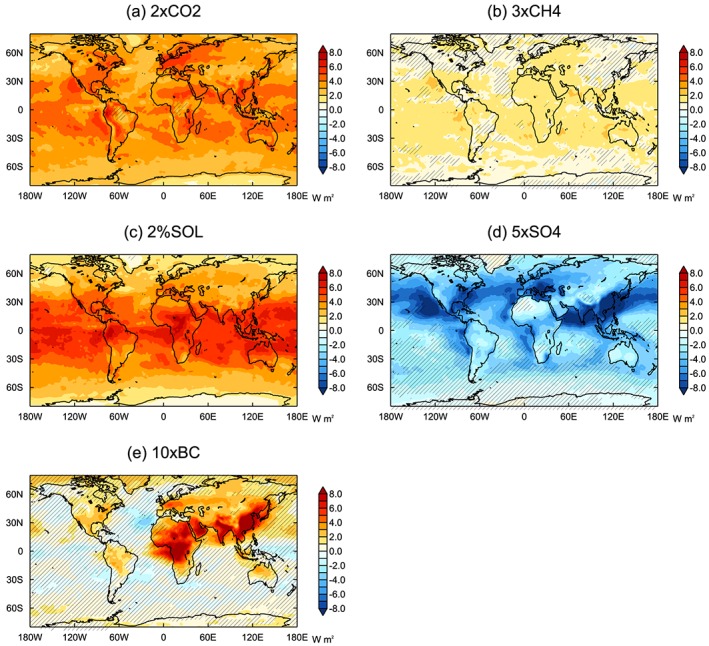
Multimodel mean *ERF*_*sst*_ distributions of core PDRMIP experiments. Hatching shows where the multimodel mean is less than the intermodel standard deviation.

The *ERF*_*sst*_ spatial pattern differs significantly between the different forcing agents (Figure 2), which could affect the efficacies (Forster et al., 2000; Hansen et al., 2005; Shindell, 2014). The forcing due to 2×CO2 and 3×CH4 is fairly homogeneous across the globe, with a slightly greater magnitude in the tropics. 2%SOL forcing exhibits a larger meridional gradient, with stronger forcing in the tropics. The aerosol perturbations produce more inhomogeneous forcing patterns. Due to the short atmospheric lifetime of aerosols, the forcing is mainly concentrated in the Northern Hemisphere close to the emission sources. For the aerosol emission‐based models, 5×SO4 *ERF*_*sst*_ is more hemispherically asymmetric, with the forcing more strongly concentrated in the Northern Hemisphere (Figure S1). The 10×BC forcing is mainly concentrated over Africa and southern Asia, and the 5×SO4 forcing is strongest over southern Asia, Europe, the North Pacific, and North Atlantic. *ERF*_*sst*_ is spatially similar for the emission‐ and concentration‐based models (Figure S1).

### 3.2 Efficacies Based on IRF and *RF*_*strat*_

**Figure 3:**
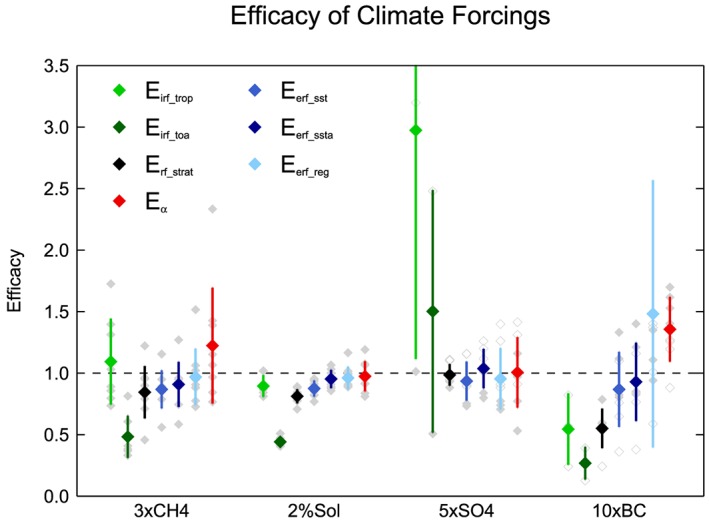
Global mean *E*_*irf_trop*_, *E*_*irf_toa*_, *E*_*rf_strat*_, *E*_*erf_reg*_, *E*_*erf_sst*_, *E*_*erf_ssta*_, and *E*_*α*_ calculated for PDRMIP forcing experiments. Colored diamonds denote the multimodel mean, and error bars show the intermodel standard deviation. Individual model results are represented by gray diamonds. Hollow gray diamonds denote models which perturbed emissions rather than concentrations in the aerosol experiments. One model result lies off the top of the scale for 5×SO4 *E*_*irf_trop*_ (MIROC‐SPRINTARS) and 10×BC *E*_*erf_r*_ (HadGEM3).

The global mean efficacies for 3×CH4, 5×SO4, 10×BC, and 2%SOL are shown in Figure 3 and Table 1. There is considerable variation between the different efficacy definitions. Both *E*_*irf_toa*_ and *E*_*irf_trop*_ vary considerably between the different forcing agents. *E*_*irf_toa*_ is substantially less than 1 for 3×CH4, 2%SOL, and 10×BC, with multimodel mean values of 0.48, 0.44, and 0.27, respectively, and relatively higher for 5×SO4 with a very large model spread, ranging from 0.51 to 2.5. *E*_*irf_trop*_ is close to one for 3×CH4 and 2%SOL but has multimodel mean values of 0.55 for 10×BC and 3.0 for 5×SO4, with considerable model spread. Hansen et al. (2005) similarly found BC to have a low efficacy when based on IRF (at the tropopause) but found methane to have an efficacy greater than 1. Consistent with Hansen et al. (2005), we find that across the PDRMIP models IRF is a poor predictor of the GSAT response to different forcing agents whether computed at the TOA or tropopause.

### 3.3 Efficacies Within the ERF Framework

When computed using ERF, multimodel mean efficacies (*E*_*erf_sst*_, *E*_*erf_ssta*_, and *E*_*erf_reg*_) are generally much closer to unity across the different perturbation experiments (Figure 3 and Table 1). This demonstrates that ERF is a better predictor of the climate response to different forcing agents. Under the IRF framework, short timescale tropospheric and stratospheric adjustment processes are considered part of the climate response and thus affect the efficacies. In contrast, efficacies based on ERF only describe differences in feedback strength to perturbations, as fast adjustments are accounted for in the forcing. The high multimodel mean *E*_*erf_reg*_ of 1.48 for 10×BC can be mainly attributed to one outlying model, HadGEM3, which has an *E*_*erf_reg*_ of 4.1, largely due to a small *ERF*_*reg*_ of 0.18 W m^−2^.

*ERF*_*ssta*_ and *ERF*_*sst*_ are generally the best predictors (i.e., efficacy closest to unity) of the GSAT response to forcing across the PDRMIP models and forcing agents. The multimodel mean *E*_*erf_ssta*_ is systematically marginally closer to unity than *E*_*erf_sst*_. This is due to the proportionately greater difference between *ERF*_*sst*_ and *ERF*_*ssta*_ for 2×CO2 compared to the other perturbations. The greater difference may be due to the physiological effect of CO_2_ on vegetation. Increasing atmospheric CO_2_ concentration reduces stomatal opening, which can reduce evapotranspiration and enhance surface warming (Andrews et al., 2010; Boucher et al., 2009; Cao et al., 2010; Field et al., 1995; Pu & Dickinson, 2014). As a result, CO_2_ drives greater land surface warming than any other driver in the fSST simulations used to compute *ERF*_*sst*_. Therefore, accounting for land surface temperature‐driven feedbacks in the fSST simulations using radiative kernels enables a slightly better prediction of the climate response to forcing.

Multimodel mean *E*_*erf_ssta*_ and *E*_*erf_sst*_ are both close to one for 5×SO4 (0.98 and 0.94, respectively) and slightly less than 1 for 10×BC (both 0.87). Marvel et al. (2015) similarly found that the efficacy of tropospheric aerosols is closer to one when using ERF, rather than IRF. However, the PDRMIP results are in contrast to previous work which suggests that tropospheric aerosols have an efficacy around 40–50% higher than CO_2_ over the historical period (Rotstayn et al., 2015; Shindell, 2014; Shindell et al., 2015). The high efficacy of aerosols has mainly been attributed to the inhomogeneous pattern of the forcing (Shindell, 2014). Over the historical period tropospheric aerosol forcing has been concentrated in the Northern Hemisphere where the temperature response may be enhanced by strong positive feedbacks at high latitudes (Pithan & Mauritsen, 2014), less moisture availability over land (Joshi et al., 2008), and a faster transient response. However, despite the fact that the forcing is predominantly concentrated in the Northern Hemisphere in the 5×SO4 and 10×BC simulations (Figure 2), an enhanced GSAT response is not evident in most models.

**Figure 4:**
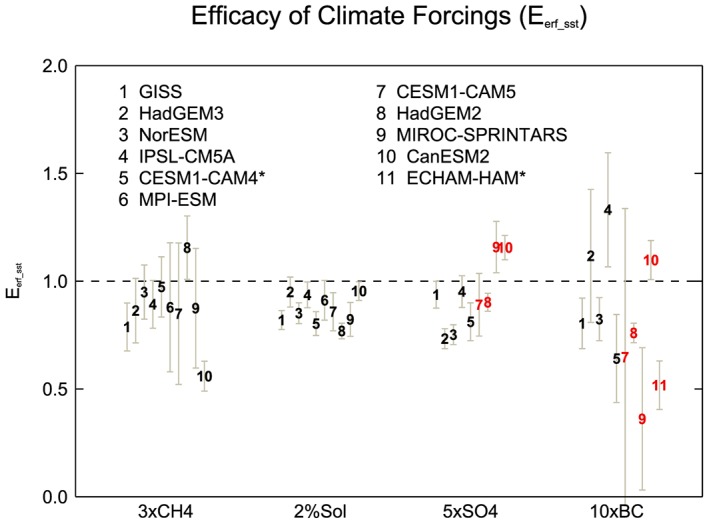
*E*_*erf_sst*_ estimates for each PDRMIP model. Error bars denote the 5–95% confidence interval based on the interannual variability of the control run. Models which perturbed emissions rather than concentrations in the aerosol experiments are shown in red. CESM1‐CAM4 and ECHAM‐HAM are run with a slab ocean.

*E*_*erf_sst*_ for each model and experiment is shown in Figure 4, along with 5–95% confidence intervals. There is considerable intermodel spread in the 5×SO4 and 10×BC efficacies. For all concentration‐based models, the efficacy of 5×SO4 is less than one (0.73–0.95), whereas the emission‐based models exhibit efficacies both higher and lower than one (0.89–1.16). How aerosol emissions translate into atmospheric burden, and hence forcing, may introduce a larger degree of variability in efficacy results than indicated from the concentration‐based experiments. Both concentration‐ and emission‐based models produce a large intermodel spread in efficacies for 10×BC, ranging from 0.36 to 1.33 (Figure 4). The large uncertainties can mainly be attributed to a small TOA forcing and weak GSAT response (Stjern et al., 2017). In addition, both the forcing and climate response to BC are dependent on the vertical profile of the BC aerosols (Ban‐Weiss et al., 2012); therefore, TOA ERF may not be a useful predictor of the GSAT response. Looking across the model ensemble as a whole there is the efficacy of sulfate or BC aerosols that varies substantially leaving it unclear whether it significantly differs from unity.

Methane has an *E*_*erf_sst*_ of less than one in all models except one, ranging from 0.56 to 1.15. However, the uncertainty is generally large, and for seven of the 10 models, an efficacy of one lies within the confidence intervals. Hansen et al. (2005) found methane to have an efficacy close to unity (0.87–1.08) when based on ERF, but more recently a lower estimate of 0.81 was obtained (Modak et al., 2018). Modak et al. (2018) attribute the lower efficacy to differing rapid adjustments of upper tropospheric temperature, lower stratospheric temperature, and clouds, which then affect the slow temperature response and lapse rate feedback.

**Figure 5:**
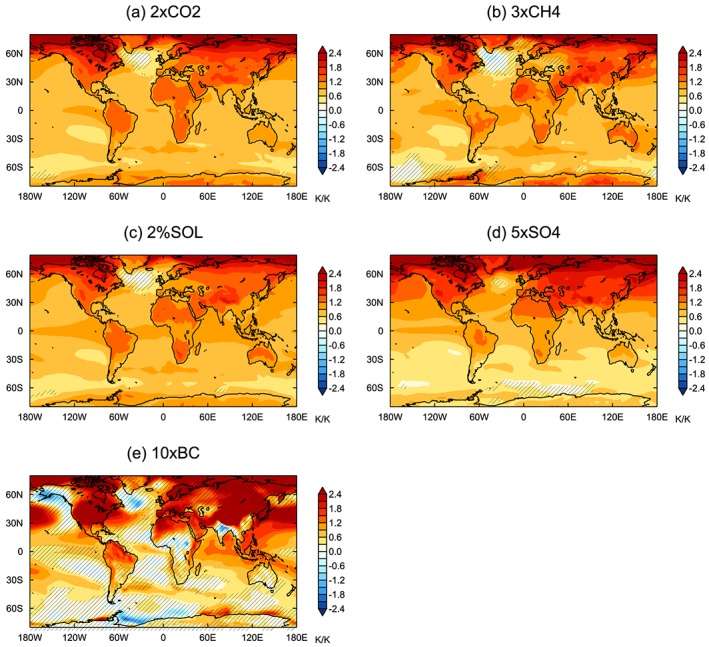
Multimodel mean surface temperature response for core PDRMIP forcing experiments (years 81–100 of coupled runs), normalized by the GSAT response. Hatching shows where the multimodel mean is less than the intermodel standard deviation.

**Figure 6:**
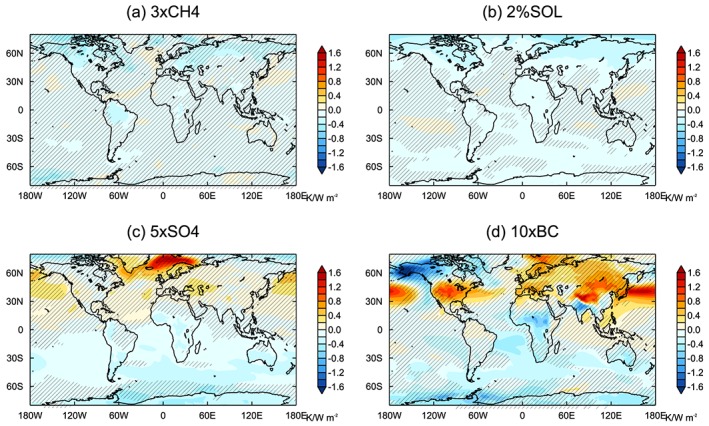
Multimodel mean difference in normalized surface temperature responses for each of the core PDRMIP perturbation experiments relative to temperature response to 2×CO2. Temperature responses are normalized by *ERF*_*sst*_. Hatching shows where the multimodel mean is less than the intermodel standard deviation.

*E*_*erf_sst*_ is slightly less than one across models for 2%SOL, ranging from 0.77 to 0.95. Previous studies generally find efficacies of less than one for solar forcing (Forster et al., 2000; Hansen et al., 2005; Marvel et al., 2015; Modak et al., 2016). The weaker efficacy may be due to the meridional structure of solar forcing (Figure 2), which leads to less warming at high latitudes (Figures 5 and 6). Warming in the tropics is more efficiently damped by radiative feedbacks (Kang & Xie, 2014). Modak et al. (2016) also suggest that tropospheric cloud adjustments and stratospheric warming lead to the weaker efficacy for solar forcing.

### 3.4 Spatial Pattern and Vertical Structure of Temperature Response

We next look at how the surface temperature changes are manifested spatially. Figure 5 shows the spatial pattern of surface temperature change normalized by GSAT change for each perturbation. Despite the large spatial differences in forcing patterns for the different drivers, the temperature responses project onto broadly similar spatial patterns, with greater changes occurring in the Northern Hemisphere at higher latitudes and over land regions. The hemispheric asymmetry is strongest for the aerosol perturbations, which is consistent with the forcing being concentrated in the Northern Hemisphere (Figure 2).

Figure 6 displays the surface temperature response normalized by *ERF*_*sst*_ for each perturbation relative to the 2×CO2 surface air temperature response. The larger hemispheric asymmetry in surface temperature change for the aerosol perturbations is evident. The temperature response is robustly weaker over most of the Southern Hemisphere for 5×SO4 and 10×BC, where the local forcing is comparatively small. In the Northern Hemisphere there are various regions where surface temperature is more sensitive to the aerosol perturbations. In response to 5×SO4, surface temperature is notably more sensitive over the Norwegian and Barents Seas than for 2×CO2. The higher temperature sensitivity in the northern midlatitudes for 5×SO4 appears to be more prominent in the emission‐based models (Figure S2). However, given the different formulations and different aerosol concentrations within the individual emission‐based models, it is difficult to determine what factors would lead to this effect.

In response to 10×BC, surface temperature is more sensitive than for 2×CO2 in western Asia, the northern Pacific, North America, and the Mediterranean. Interestingly, the regions of higher temperature sensitivity to the aerosol forcing do not necessarily occur in regions where the emissions and forcing are greatest, nor where they are comparatively larger than for 2×CO2.

The 3×CH4 perturbation leads to a very similar spatial pattern of warming to 2×CO2. There are some locations over land where the 3×CH4 temperature response is robustly weaker than for 2×CO2, which coincide with the Amazon, central African, and boreal forests. In these densely vegetated regions, the physiological effect of CO_2_ on plants can strongly reduce evapotranspiration and therefore enhance warming (Andrews et al., 2010; Cao et al., 2010). Modeling studies suggest that the physiological effect enhances land mean surface warming in response to CO_2_ by around 15% (Boucher et al., 2009; Cao et al., 2010; Pu & Dickinson, 2014). This may also contribute to the robustly weaker temperature response to 2%SOL over many land regions relative to 2×CO2. The temperature response to 2%SOL is robustly weaker than for 2×CO2 at high latitudes in the Northern Hemisphere which is likely due to the weaker forcing there relative to the tropics for 2%SOL.

**Figure 7:**
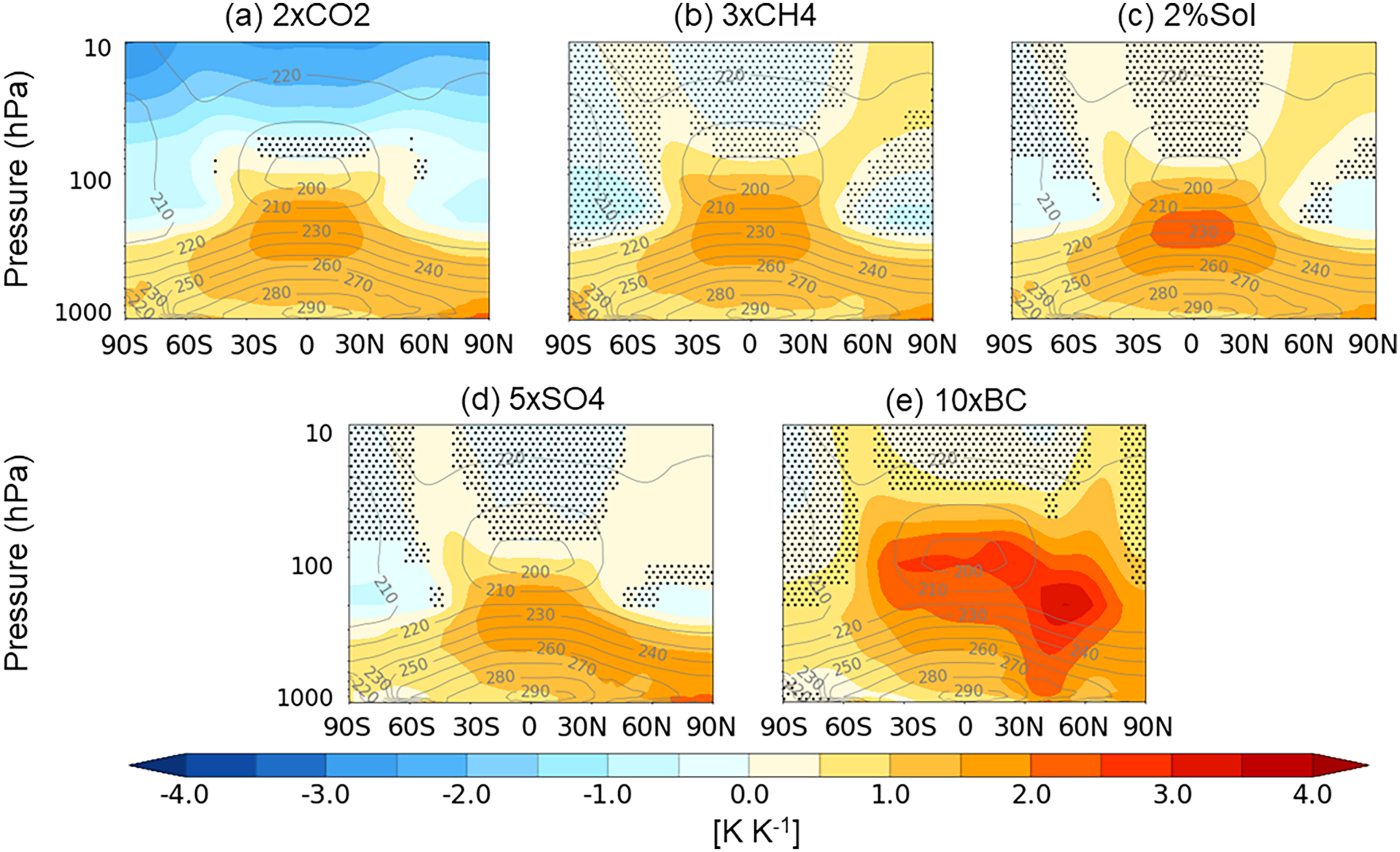
PDRMIP multimodel zonal mean temperature response to forcing normalized by GSAT change. The gray contours show the multimodel mean zonal temperature climatology. The stippling shows where the MMM difference is less than ±1 standard deviation of the intermodel spread.

We now consider the vertical structure of the temperature responses in the PDRMIP experiments, as shown in Figure 7. Except for the 10×BC experiment, each of the forcings exhibits broadly similar large‐scale patterns of tropospheric temperature change, with amplification in the tropical upper‐troposphere and near‐surface amplification at the poles, particularly in the Arctic. The amplified tropical upper tropospheric temperature change is a robust feature of the response to greenhouse gases in global climate models (Ceppi & Shepherd, 2017; Gillett et al., 2000; Rotstayn et al., 2013; Santer et al., 2013) with a similar trend also evident in observations since the late 1950s (Santer et al., 2017; Vinnikov et al., 1996). Radiative forcing in the tropics produces less surface heating than the poles as more energy is used to increase evaporation. This leads to increased latent heat release in the upper troposphere, and the vertical temperature profile adjusts toward the moist adiabatic lapse rate (Mitchell et al., 1990; Wilson & Mitchell, 1987).

The tropospheric temperature response to aerosols is less hemispherically symmetric, consistent with the asymmetric nature of the aerosol forcing (Figure 2) and surface temperature response (Figure 5). 10×BC in particular drives a noticeably different pattern of atmospheric temperature change to the other drivers, with strong heating near the tropopause. Unlike most other drivers of climate change, BC affects atmospheric temperature predominantly through rapid adjustments rather than through surface temperature mediated processes (Stjern et al., 2017). BC strongly absorbs shortwave radiation, directly heating the atmosphere without strongly warming the surface, which can account for the qualitatively different vertical profile of warming.

In the stratosphere, 2×CO2 is the only driver that induces radiative cooling across the globe. 2%SOL induces stratospheric heating at most latitudes owing to the increased absorption of ultraviolet radiation by ozone. The one exception is HadGEM3 which simulates a relatively large (~75%) increase in stratospheric water vapor in the 2%SOL experiment, which offsets the shortwave‐driven stratospheric heating (not shown). In 3×CH4 and 5×SO4 the stratospheric temperature changes show regions of heating and cooling, which are indicative of changes in the large‐scale stratospheric overturning circulation. However, most of the PDRMIP models do not fully resolve the stratosphere, which may limit their ability to capture changes in stratospheric circulation (Charlton‐perez et al., 2013).

### 3.5 Regional Aerosol Perturbations

**Figure 8:**
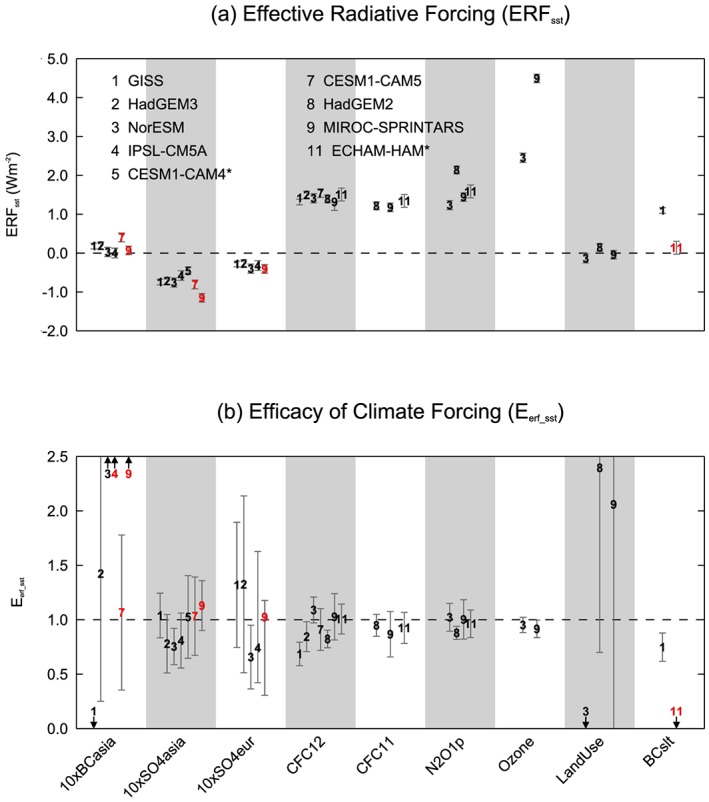
*ERF*_*sst*_ (top) and *E*_*erf_sst*_ (bottom) of each model for global and regional forcing experiments performed by a subset of PDRMIP models. The error bars denote the 5–95% confidence interval based on the interannual variability of the control run. Models which perturbed emissions rather than concentrations in the aerosol experiments are shown in red. CESM1‐CAM4 and ECHAM‐HAM are run with a slab ocean.

To investigate whether the location of aerosol forcing impacts on its efficacy, we analyze the three regional PDRMIP experiments: 10×BCasia, 10×SO4asia, and 10×SO4eur (see Table S2). Global mean *ERF*_*sst*_ results for the regional simulations are shown in Figure 8a and Table S3. As expected,10×BCasia produces strong forcing over Asia but globally averaged the forcing is small, meaning a robust efficacy calculation cannot be performed. 10×SO4asia produces a fairly large negative global mean *ERF*_*sst*_ ranging from −0.46 to −1.15 W m^−2^, and 10×SO4eur produces a smaller negative *ERF*_*sst*_ ranging from −0.27 to −0.41 W m^−2^.

Comparing the two regional sulfate perturbation experiments, there is no clear difference in efficacies across the models due to the location of the forcing (Figure 8b). *E*_*erf_sst*_ ranges from 0.66 to 1.33 for 10×SO4eur, and 0.75 to 1.13 for 10×SO4asia, showing a similarly large intermodel spread to the global 5×SO4 experiment. Liu et al. (2018) also noted that regional and global sulfate aerosol forcing drives a similar GSAT response to CO_2_ forcing. Previous studies, mainly based on the GISS model, suggest that the concentration of aerosol forcing at high latitudes may enhance its efficacy (Shindell et al., 2015; Shindell & Faluvegi, 2009). Kasoar et al. (2018) also found that the GSAT response to sulfate aerosol forcing was sensitive to the location of the source region. Similarly, in our analysis both GISS and HadGEM3 have higher efficacies for 10×SO4eur (1.32 and 1.33, respectively) than for 10×SO4asia (1.04 and 0.78, respectively). However, this result is not robust across the PDRMIP models, with no other model exhibiting a higher sensitivity to aerosol forcing over Europe than over Asia. In addition, the 10×SO4asia and 10×SO4eur efficacy results are not significantly different from unity for any of the models except NorESM. In five out of the seven models, concentrations were perturbed rather than emissions. Incorporating the models' own aerosol schemes to determine the atmospheric burden would likely introduce further uncertainty and model spread not seen in this analysis.

**Figure 9:**
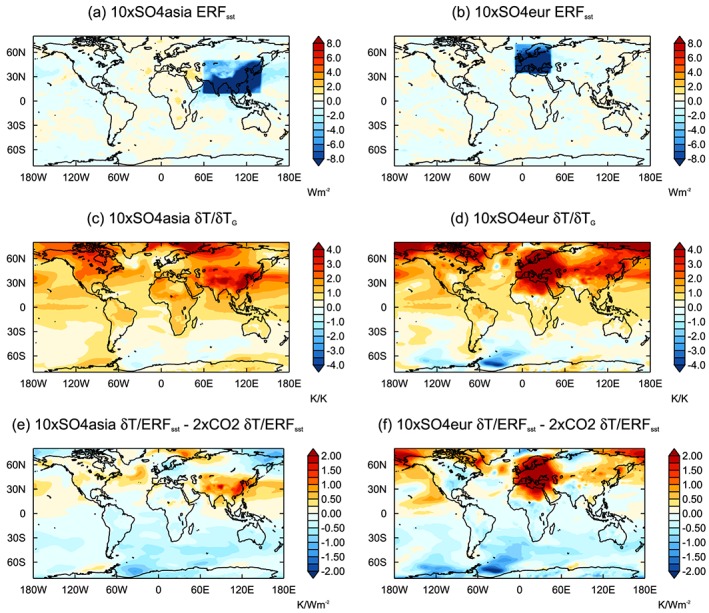
Multimodel mean *ERF*_*sst*_ for (a) 10×SO4asia and (b) 10×SO4eur. Multimodel mean surface temperature response normalized by the GSAT response for (c) 10×SO4asia and (d) 10×SO4eur. Multimodel mean difference in normalized surface temperature response relative to 2×CO2 for (e) 10×SO_4_asia and (f) 10×SO_4_eur. The temperature response is normalized by *ERF*_*sst*_.

The two regional sulfate experiments drive a similar hemispheric asymmetry in surface temperature change (Figure 9) as seen for the global perturbation (Figure 5). Surface temperature responds more strongly across the Northern Hemisphere than the Southern Hemisphere in both 10×SO4asia and 10×SO4eur, even in locations far from the forcing region. The strongest cooling occurs locally to the region of forcing. Similarly, Kasoar et al. (2018) found that when SO_2_ emissions are removed from specific regions, the temperature response does tend to be strongest local to the forcing, but there is also a common broad pattern response regardless of forcing location. The particularly strong cooling over Europe for 10×SO4eur is consistent with high‐latitude forcing being less effectively damped by radiation (Kang & Xie, 2014).

### 3.6 Efficacy of Other Forcing Agents

Using a subset of the PDRMIP models, a number of further forcing scenarios were analyzed to determine the efficacies of halocarbons, tropospheric ozone, land use change, and BC with a shorter lifetime (Figure 8 and Table S3). The spatial pattern of *ERF*_*sst*_ is shown in Figure S3. *E*_*erf_sst*_ for CFCs (CFC‐12 and CFC‐11) is generally close to or slightly less than one. For CFC‐11 the models have an *E*_*erf_sst*_ slightly smaller than unity, with values ranging from 0.87 to 0.95. However, this smaller efficacy is not robust as the 5–95% uncertainty range for each model still includes unity. CFC‐12 exhibits more model spread with *E*_*erf_sst*_ ranging from 0.69 to 1.09. *E*_*erf_sst*_ for N_2_O ranges from 0.88 to 1.02, with only one model (HadGEM2) having an efficacy significantly different from one. This is consistent with Hansen et al. (2005) who also found that CFCs and N_2_O have an efficacy close to one when computed using ERF.

Ozone forcing in NorESM and MIROC‐SPRINTARS produces a global mean efficacy of 0.95 and 0.92, respectively, which is larger than previous results (Hansen et al., 2005; Marvel et al., 2015; Yoshimori & Broccoli, 2008). Spatially, the ozone forcing produces a more hemispherically asymmetric warming pattern than 2×CO2 with a higher sensitivity in the Northern Hemisphere (Figure S4). The land use change experiment produces a very small global mean *ERF*_*sst*_ ranging from −0.14 to 0.15 W m^−2^ across models, resulting in highly uncertain efficacies. Shortening the lifetime of BC in GISS appears to make little difference to the efficacy. For ECHAM‐HAM shortening BC lifetime produces a very small *ERF*_*sst*_ resulting in a highly uncertain efficacy.

### 3.7 Climate Feedback Parameter

It is possible to estimate forcing efficacies based on the climate feedback parameter (*E*_*α*_ in Figure 3 and Table 1), under the assumption that the climate feedback parameter remains constant in time. However, various studies have shown that the climate feedback parameter in response to an abrupt increase in CO_2_ is not constant and tends to change such that the climate sensitivity increases as equilibrium is approached (Andrews et al., 2015; Armour et al., 2013; Gregory, 2004; Haugstad et al., 2017; Rose & Rayborn, 2016; Rugenstein et al., 2016; Winton et al., 2010). This is generally attributed to evolving patterns of surface temperature change, which actuate different local feedbacks on different timescales (Andrews et al., 2015, 2018; Rugenstein et al., 2016). It is therefore important to understand the time evolution of the climate feedback parameter and whether this is affected by the changing mix of time‐evolving forcings (Marvel et al., 2015).

The climate feedback parameters computed using the first 20 years (*α_*20) and final 80 years (*α_*80) of the five core PDRMIP simulations are shown in Table 1. Across forcing experiments the feedback parameter is higher in the first 20 years and has a tendency to decrease through the runs in the majority of models. The time evolution of the feedback parameter for the 5×SO4 simulation is notably different to 2×CO2. The feedback parameter in response to sulfate forcing tends to exhibit a larger reduction in magnitude through the simulations, with a multimodel mean of −1.51 ± 0.85 W m^−2^/K for the first 20 years decreasing to −0.80 ± 0.36 W m^−2^/K in the final 80 years. This is in comparison to −1.27 ± 0.52 W m^−2^/K and −1.03 ± 0.57 W m^−2^/K for 2×CO2. Therefore, the effective climate sensitivity in terms of temperature change per unit forcing computed from the first 20 years of simulations is lower for sulfate than for CO_2_. This is in contrast to recent studies which suggest that the temperature change per unit forcing of tropospheric aerosols could be higher than for CO_2_ within transient simulations (Shindell, 2014; Shindell et al., 2015).

### 3.8 Impact of Efficacies on Historical Temperature Change

**Figure 10:**
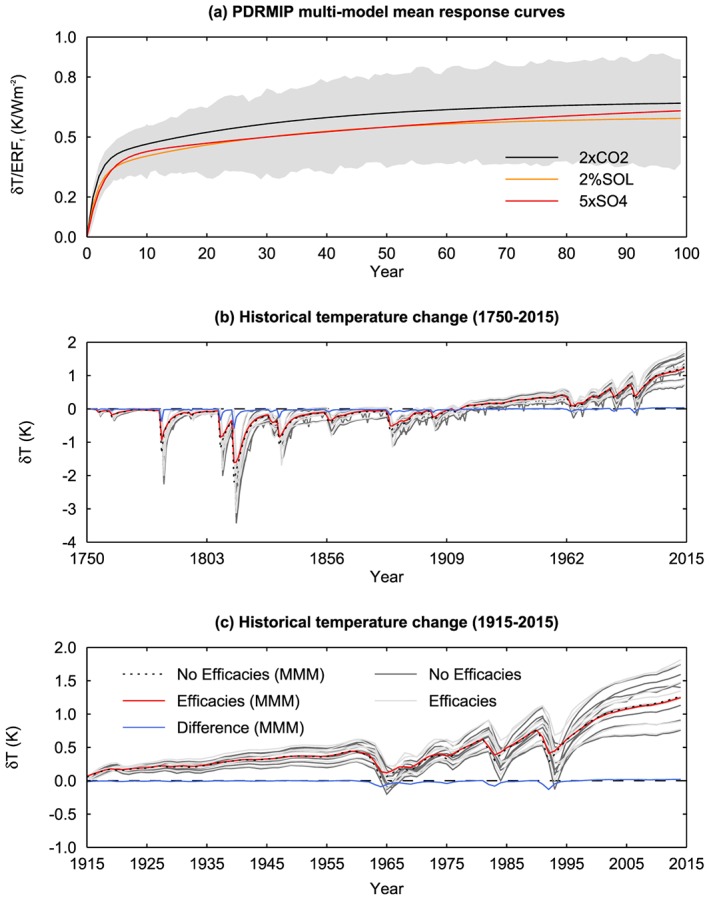
Panel (a) shows the multimodel mean GSAT response curves as a function of time for 2×CO_2_, 5×SO_4_, and 2%SOL normalized by *ERF*_*sst*_. The intermodel standard deviation is shown in gray. Panel (b) shows the multimodel mean historical GSAT change computed using the impulse response model described in section 2.5 not accounting for efficacies (red) and accounting for efficacies (black dotted). The difference is shown in blue. Individual model results not accounting for efficacies are shown by dark gray lines GSAT and accounting for efficacies by light gray lines. Panel (c) shows the same as panel (b) for the period 1915–2014 and therefore includes no extrapolation of the response curves beyond the 100 years of PDRMIP data.

Figure 10a shows the multimodel mean GSAT response curves normalized by *ERF*_*sst*_ for 2×CO2, 5×SO4, and 2%SOL. It can be seen that in the early stages of the integrations the multimodel mean GSAT response to sulfate lags behind that of CO_2_, before converging again later in the simulations. The lag in GSAT change is due to subdued warming in the Southern Hemisphere (Figure S5). The response curve for 10×BC (Figure S6) is similar to the 5×SO4 response, but with proportionately larger uncertainties owing to the overall smaller GSAT response to BC in the experiments. 2%SOL drives a smaller GSAT response than 2×CO2 throughout the simulations. This is due to relatively weaker warming in the Northern Hemisphere (Figure S5), which is a manifestation of the weaker polar amplification seen in Figure 6. The 3×CH4 response curve (Figure S6) is similar to the 2%SOL. The differences in the multimodel mean response curves for each forcing type, however, are substantially smaller than the intermodel spread (gray shading in Figure 10a shows the standard deviation of intermodel spread).

We construct a simple model to estimate GSAT change in response to historical TOA forcing based on the PDRMIP response curves (see section 2.5). To investigate the importance of efficacies for interpreting historical trends, we use the simple model to compute GSAT change using the same response curve (2×CO2) for all forcing agents (Figures 10b and 10c, black lines) as well as using distinct response curves for CO_2_, CH_4_, insolation, total aerosol (represented by the 5×SO4 response curve), and volcanic forcing (represented by the 2%SOL response curve). All other remaining forcings are attributed the 2×CO2 response curve. This is done using both the multimodel mean response curves and for each individual model (see Figure S7). Based on the multimodel mean response curves, accounting for the different multimodel mean efficacies has very little impact on the historical GSAT trend with the largest differences found following volcanic eruptions (Figures 10b and 10c, difference shown in blue).

Accounting for efficacies has a larger effect for some individual models, in particular CanESM2. However, for many of the models, accounting for efficacies has a very small effect on the historical GSAT trend. Monte Carlo simulations (10,000 realizations) were performed to understand how uncertainty in the efficacy affects simulation of historic temperatures in the simple model. Each efficacy was randomly sampled across the PDRMIP model range for each forcing agent separately, and these sampled efficacies were used to scale the forcing‐response curves in the simple model to construct an overall GSAT trend (see Figures 10b and 10c). The simple model gives a best estimate of GSAT change over 1915–2015 of 1.2 ± 0.17 K, with the 5–95% range quoted due to the PDRMIP model efficacy variations. This GSAT trend was used to derive historic climate sensitivity using the approach taken in many papers (e.g., Forster, 2016; Marvel et al., 2015) of estimating *α* from equation (1) and equilibrium climate sensitivity (ECS) from *F*_×CO2_ */α*. The 1.2 ± 0.17 K GSAT trend estimate over 1915–2015 was combined with estimates of *F*, *N*, and *F*_×CO2_ of 2.2, 0.6, and 3.7 W m^−2^, respectively, from Forster et al. (2016). This gives an ECS from the historical of 2.91 ± 0.4 K, with the 5% to 95% uncertainties due to possible efficacy variation of roughly ±14%; this can be compared to a nonefficacy best estimate ECS of 2.88 K. The difference is considerably smaller than the 1.0 K uplift from 2.0 to 3.0 K suggested by Marvel et al. (2015), when ERF efficacy was accounted for their model.

Studies which have found significant differences in efficacies (Marvel et al., 2015; Shindell, 2014) which could affect climate sensitivity have tended to be based on historical transient simulations, rather than step‐change experiments as used here. The differing methodologies of computing forcing and efficacies could contribute to differing results (Lewis and Curry, 2018). In particular, the version of the GISS model used here had no aerosol indirect effects or interactive chemistry so is not directly comparable to some earlier analyses. It should be noted that the aerosol perturbations used in this study were very large, and it is assumed that the temperature response scales linearly with forcing strength, so that the efficacies computed are applicable for the historical period. Further studies would be needed to explore this, and long model integrations would likely be needed to improve the signal to noise compared to that found here and in earlier studies.

## 4 Conclusions

Quantifying the effectiveness of different forcing agents in driving surface temperature change is important for understanding the real‐world climate sensitivity and for projecting future levels of surface warming for different emission pathways. In this study we have presented a systematic multimodel analysis of the efficacies of a wide range of different climatic drivers, in particular methane, insolation, sulfate, and BC aerosols. Consistent with previous studies, we find that efficacies calculated using IRF vary significantly across drivers. This is because rapid atmospheric adjustments to both CO_2_ (e.g., stratospheric temperatures) and other drivers (e.g., cloud adjustments) are not captured by the IRF concept and are instead attributed as part of the long‐term climate response. When computed based on ERF, which does account for rapid adjustments, the multimodel mean efficacies are much closer to unity across drivers. However, there remains a large intermodel spread, in particular in response to sulfate and BC forcing.

We find that ERF definitions based on fixed SST experiments (*ERF*_*sst*_ and *ERF*_*ssta*_) are the best predictors of the climate response (efficacies closest to one) across different forcing agents. Efficacies are marginally closer to one when *ERF*_*sst*_ is adjusted to take into account land surface temperature change using radiative kernels. *E*_*erf_sst*_ is less than one across all models for solar forcing (0.77–0.95). Multimodel mean *E*_*erf_sst*_ is slightly less than one for methane, sulfate, and BC forcing, but there is a greater intermodel spread (3×CH4: 0.56–1.15, 5×SO4: 0.73–1.16, and 10×BC: 0.36–1.33). Spatially, there are some differences in the surface temperature response to different forcings. The aerosol experiments exhibit weaker temperature changes in the Southern Hemisphere and stronger responses in some regions of the Northern Hemisphere relative to CO_2_. However, the regions where surface temperature is more sensitive to aerosol forcing are *not* generally where the forcing is strongest. Sulfate forcing in Europe has a slightly higher efficacy than forcing in Asia in the multimodel mean. However, the model spread is large, and there is no robust indication across models that the efficacies differ from unity. The considerable model spread in aerosol efficacies found in this study indicates that previous findings on aerosol efficacies relying on single model studies may not be robust.

The radiative feedbacks in response to GSAT change are generally not constant and evolve through time. Across the five core experiments the climate feedback parameter tends to decrease through the simulations (i.e., sensitivity increases). This agrees with previous work on the evolution of model response to CO_2_ (e.g., Armour, 2017). The time evolution of the climate feedback parameter differs slightly between forcing experiments. In response to sulfate forcing the change in the climate feedback parameter is particularly large. The GSAT response to 5×SO4 initially lags behind the response to 2×CO2 due to a subdued southern hemispheric response. However, as the climate feedback parameter reduces later in the simulation, GSAT begins to change faster in response to sulfate forcing than for CO_2_.

Despite evidence of a forcing‐response relationship that depends on forcing type, we find that overall there is not a robust indication that accounting for the different GSAT response curves gives an appreciably different GSAT evolution over the historical period than assuming that the response curves of all forcing agents follow that of CO_2_. This result is valid for forcing‐response curves normalized by ERF. Forcing definitions that do not account for rapid adjustments do exhibit considerable efficacy variation. For example, using *RF*_*strat*_ to construct historic GSAT would overestimate the role of BC if efficacies were not accounted for.

Our results indicate that ERF efficacies could be close enough to one that they will not significantly affect climate sensitivity estimates derived from the historical period temperature trend. However, considerable uncertainty in our computed efficacies remains. A role for efficacy variation in historical temperature evolution (of ±14%) cannot be ruled out, with a similar relative effect on derived estimates of ECS (±0.4 K), indicating that it could still be important to consider. Future work should focus on understanding the physical differences for the model spread to determine where variations are robust or more an issue of signal to noise.

## Supporting information

Supporting Information S1
