# Supplementary material for: Efficacy of Climate Forcings in PDRMIP Models
Source: J Geophys Res Atmos. 2019 Dec 11;124(23):12824–44. doi: 10.1029/2019JD030581 (PMC6988499; doi:10.1029/2019JD030581)
Supplement: Supplementary file 1 — Supporting Information S1 [file JGRD-124-12824-s001.docx]

**Supplementary Materials: Efficacy of climate forcings in PDRMIP models**

T. B. Richardson^1^, P. M. Forster^1^, C. J. Smith^1^, A. C. Maycock^1^, T. Wood^1^, T. Andrews^2^, O. Boucher^3^, G. Faluvegi^4^, D. Fläschner^5^, Ø. Hodnebrog^6^, M. Kasoar^7^, A. Kirkevåg^8^, J.-F. Lamarque^9^, J. Mülmenstädt^10^, G. Myhre^6^, D. Olivié^8^, R. W. Portmann^11^, B. H. Samset^6^, D. Shawki^7^, D. Shindell^12^, P. Stier^13^, T. Takemura^14^, A. Voulgarakis^7^, D. Watson-Parris^13^

Table S1: PDRMIP model details.

| **Model** | **Version** | **Ocean Setup** | **Aerosol and ChemistrySetup** | **Baseline** |
| --- | --- | --- | --- | --- |
| CanESM2 | 2010 | Coupled Ocean | Emissions based. *Full microphysics for aerosol-cloud interactions*. No oxidation chemistry included for CH_4_-ozone or CH_4_-stratospheric water vapor | Present-day |
| CESM1-CAM4 | 1.0.3 | Slab Ocean | Prescribed concentrations of aerosol and greenhouse gases. *No aerosol microphysics effects*. No oxidation chemistry included for CH_4_-ozone or CH_4_-stratospheric water vapor | Present-day |
| CESM1-CAM5 | 1.1.2 | Coupled Ocean | Emissions based. *Full aerosol microphysics.* No oxidation chemistry included for CH_4_-ozone or CH_4_-stratospheric water vapor | Present-day |
| ECHAM-HAM | ECHAM6.3-HAM2.3 | Slab Ocean | Emissions based.  *Full aerosol microphysics*. CH_4_-stratospheric water vapor parameterised with no other interactive chemistry. | Present-day |
| GISS | E2-R | Coupled Ocean | Prescribed concentrations of aerosol and greenhouse gases. *No aerosol microphysics effects.* CH_4_-stratospheric water vapor chemistry parameterisation included with no other interactive chemistry | Present-day |
| HadGEM2 | 6.6.3 | Coupled Ocean | Emissions based  *Full microphysics for aerosol-cloud interactions*. No oxidation chemistry included for CH_4_-ozone or CH_4_-stratospheric water vapor | Pre-industrial |
| HadGEM3 | GA 4.0 | Coupled Ocean | Prescribed concentrations of aerosol and greenhouse gases. *No microphysics for black carbon or dust. Microphysics of aerosol cloud interaction included for other species through cloud condensation nuclei prescribed change*. CH_4_-stratospheric water vapor chemistry parameterisation included with no other interactive chemistry | Present-day |
| IPSL-CM5A | CMIP5 | Coupled Ocean | Prescribed concentrations of aerosol and greenhouse gases. *Aerosol microphysics for Twomey effect included*. No oxidation chemistry included for CH_4_-ozone or CH_4_-stratospheric water vapor | Present-day |
| MPI-ESM | 1.1.00p2 | Coupled Ocean | No aerosol or chemistry: *no aerosol experiments performed*, Prescribed concentrations of greenhouse gases. | Present-day |
| NorESM | 1-M | Coupled Ocean | Prescribed concentrations of aerosol and greenhouse gases. *Microphysics of aerosol cloud interaction included through cloud condensation nuclei prescribed change*. No oxidation chemistry included for CH_4_-ozone or CH_4_-stratospheric water vapor | Present-day |
| MIROC-SPRINTARS | 5.9.0 | Coupled Ocean | Emissions based. *Full microphysics for aerosol-cloud interactions*. No oxidation chemistry included for CH_4_-ozone or CH_4_-stratospheric water vapor | Present-day |

Table S2: Description of PDRMIP experiments.

| **Experiment** | **Description** |
| --- | --- |
| **Control** | All anthropogenic and natural climate forcing agents set to present day (pre-industrial for HadGEM2). |
| **2xCO2** | CO_2_ concentration doubled relative to control. |
| **3xCH4** | CH_4_ concentration tripled relative to control. |
| **10xBC** | BC concentration or emissions increased 10 times relative to control. |
| **5xSO4** | Sulphate concentration or emissions increased by 5 times relative to control. |
| **2%SOL** | Solar irradiance increased by 2%. |
| **10xBCasia** | BC concentration multiplied by 10 for Asia only. |
| **10xSO4asia** | Sulphate concentration multiplied by 10 for Asia only. |
| **10xSO4eur** | Sulphate concentration multiplied by 10 for Europe only. |
| **CFC12** | CFC-12 mixing ratio from 535ppt (control) to 5ppb. |
| **CFC11** | CFC-11 mixing ratio from 653.45ppt (control) to 5ppb. |
| **N2O1p** | N2O mixing ratio from 316ppb (control) to 1ppm. |
| **Ozone** | 5 times the tropospheric ozone distribution as used in *MacIntosh et al.* [2016]. |
| **LandUse** | Change vegetation from CMIP5 piControl base state to present-day as used in PDRMIP Control run. |
| **BCslt** | BC lifetime changed to approximately 4 days. |

**Table S3:** Multi-model mean effective radiative forcing (*ERF_sst_*), GSAT change (*ΔT*), and forcing efficacies (*E_erf_sst_*) for the regional and other experiments performed by a subset of the PDRMIP models (see Table S1 for model details). Model range is shown in brackets.

| **Experiment** | **ERF_f_ (W m^-2^)** | **ΔT (K)** | **ΔT/ERF_f_ (K/W m^-2^)** | **E_erf_f_** |
| --- | --- | --- | --- | --- |
| **10xBCasia** | 0.15 (0.002 to 0.40) | 0.15 (-0.06 to 0.30) | 7.92 (-0.32 to 36.2) | 10.8 (-0.85 to 46.3) |
| **10xSO4asia** | -0.79 (-1.15 to -0.58) | -0.44 (-0.61 to -0.29) | 0.57 (0.39 to 0.86) | 0.92 (0.75 to 1.04) |
| **10xSO4eur** | -0.34 (-0.41 to -0.27) | -0.21 (-0.39 to -0.14) | 0.67 (0.39 to 1.45) | 1.01 (0.66 to 1.33) |
| **CFC12** | 1.39 (1.21 to 1.54) | 0.82 (0.34 to 1.39) | 0.58 (0.26 to 0.93) | 0.90 (0.69 to 1.09) |
| **CFC11** | 1.19 (1.17 to 1.21) | 0.66 (0.41 to 0.91) | 0.55 (0.35 to 0.75) | 0.91 (0.87 to 0.95) |
| **N2O1p** | 1.60 (1.23 to 2.14) | 0.94 (0.58 to 1.48) | 0.57 (0.40 to 0.69) | 0.97 (0.88 to 1.02) |
| **Ozone** | 3.47 (2.45 to 4.49) | 1.52 (1.39 to 1.65) | 0.47 (0.37 to 0.57) | 0.93 ( 0.92 to 0.95) |
| **Landuse** | -0.01 (-0.14 to 0.15) | 0.12 (-0.03 to 0.28) | 0.64 (-0.81 to 1.89) | 1.03 (-1.36 to 2.40) |
| **BCslt** | 1.10 | 0.31 | 0.28 | 0.75 |

**Figure S1:** Multi-model mean *ERF_sst_* of (a, b) 10xBC and (c, d) 5xSO4 experiments for (a, c) models which perturbed emissions and (b,d) models with perturbed concentrations. Hatching denotes where the multi-model mean is less than the inter-model standard deviation.

**Figure S2:** Multi-model mean difference in normalised surface temperature responses relative to 2xCO2 for (a, b) 10xBC and (c, d) 5xSO4 experiments for (a, c) models which perturbed emissions and (b, d) models which perturbed concentrations. Temperature responses are normalised by *ERF_sst_*. Hatching denotes where the multi-model mean is less than the standard deviation.

**Figure S3:** Multi-model mean *ERF_sst_* for global and regional experiments performed by a subset of the PDRMIP models (see Table S1 and S2 for model details and experiment descriptions).

**Figure S4:** Multi-model mean difference in normalised surface temperature responses for experiments run by a subset of PDMRIP models (see Table S1 and S2 for model details and experiment descriptions). The temperature responses are normalised by *ERF_sst_*.

**Figure S5:** Multi-model mean time-evolution of (a) northern hemisphere land mean, (b) southern hemisphere land mean (c) Northern hemisphere sea mean (d) Southern hemisphere sea mean temperature responses to the core PDRMIP forcing experiments normalized by global mean *ERF_sst_*. Values are smoothed using a 5-year window.

**Figure S6:** Multi-model mean GSAT response curves normalised by *ERF_sst_* for (a) 3xCH4 and (b) 10xBC.

**Figure S7:** GSAT change for the period 1915-2015 computed using the impulse response model described in section 2.5 for each of the PDRMIP models not accounting for efficacies (dotted line) and accounting for efficacies (solid line).

**Figure S8:** Panel (a) shows the multi-model mean historical GSAT change computed using the impulse response model described in section 2.5 not accounting for efficacies (red) and accounting for efficacies (black) based on ERF_sst_ (solid lines). The difference is shown in blue. Dotted lines show the same when computed using ERF_ssta_. Panel (b) shows the same as panel (a) for the period 1915-2014, and therefore includes no extrapolation of the response curves beyond the 100 years of PDRMIP data.
